# Pharmacological evaluation and binding behavior of 4,4′-diamino-2,2′-stilbene disulfonic acid with metal ions using spectroscopic techniques

**DOI:** 10.1016/j.heliyon.2024.e34639

**Published:** 2024-07-14

**Authors:** Madeeha Shabnam, Eman A. Alabdullkarem, Muhammad Saeed Jan, Saad H. Alotaibi, Khairia Mohammed Al-Ahmary, Muhammad Ibrar, Mohamed Hussien, Asmaa E. Sherif

**Affiliations:** a Department of Chemistry, Women University, Mardan, KP, Pakistan; b Chemistry Department, College of Science, King Saud University, Riyadh, 11451, Saudi Arabia; c Department of Pharmacy, Bacha Khan University, Charsadda, 24420, KP, Pakistan; d Department of Chemistry, Turabah University College, Taif University, P.O. box 11099, Taif, 21944, Saudi Arabia; e Department of Chemistry, College of Science, University of Jeddah, Jeddah, Saudi Arabia; f Department of Chemistry, Faculty of Science, King Khalid University, P.O. box 9004, Abha, 61413, Saudi Arabia; g Department of Pharmacognosy, College of Pharmacy, Prince Sattam Bin Abdul Aziz University, Al-Kharj, 11942, Saudi Arabia; h Department of Pharmacognosy, Faculty of Pharmacy, Mansoura University, Mansoura, 35516, Egypt

**Keywords:** ABTS, AChE, BChE, COX, DPPH, LOX, MTT assay

## Abstract

Industrial and human activities contribute significantly to the environmental contamination of heavy metal ions (HMIs), which have detrimental effects on aquatic life, plants, and animals, causing major toxicological problems. The commercially available **4,4′-diamino-2,2′-stilbenedisulfonic acid (DSD)** has been playing a vital role in the detection of heavy metal ions and has significantly inhibited a variety of cancer cells in numerous field of modern science. The current investigation aimed to ensure the detection of heavy metals ions from the environment and fluorescence imaging of DSD in the treatment of cancer cells. Fluorescence and UV–Visible spectroscopic analysis was performed to sense the selective behavior of the probe DSD with several heavy metal ions, including Fe^2+^, K^1+^, Co^2+^, Ni^2+^, Zn^2+^, Cd^2+^, Pb^2+^, Mn^2+^, Sn^2+^, and Cr^3+^. Furthermore, DSD was subjected to examine enzyme inhibition such as anti-Alzheimer, anti-inflammatory, antioxidant, anticancer, and antimicrobial activities in search of multifaceted drugs. Test compounds have demonstrated dose-dependent responses in the *in-vitro* enzyme inhibition assays for acetylcholinesterase (AChE), butyrylcholinesterase (BChE), cyclooxygenase (COX), and lipoxygenase (LOX), as well as antioxidant [DPPH = 2,2-diphenyl-1-picrylhydrazyl and ABTS = 2,2′-azino-bis (3-ethylbenzothiazoline-6-sulfonic acid]. The DSD were shown to be more effective than the conventional medication galantamine in inhibiting acetylcholinesterase (AChE) and butyrylcholinesterase (BChE), with an IC_50_ value of 12.18 and 20.87 μM, which is equivalent to the standard drug. The results obtained has revealed that DSD has the potential to become an effective sensor for the detection of Sn^2+^ ions over competing metal ions due to the inhibition of photo-induced electron transfer pathway (PET). The MTT (3-(4,5-dimethylthiazol-2-yl)-2,5-diphenyltetrazoliumbromide tetrazolium) test, demonstrated that DSD had strong anticancer effects against the brain cancer cell line NIH/3T3, HeLa and MCF-7 with an IC_50_ value of 32.59, 15.31 and 96.46 μM respectively. The antimicrobial testing has shown that DSD outperforms the standard drug cefixime against *Candida albicans* and *Pseudomonas aeruginosa*, respectively. This study makes a substantial contribution to the ongoing search for efficient treatments for breast cancer.

## 1 Introduction

HMIs and persistent organic pollutants (POPs) have also been shown to be dramatically elevated in the environment as a result of rapid industrialization and human activities. These include ions created during the nuclear power generation process, such as Cr^3+^, Pb^2+^, Fe^2+^, and radionuclides. The introduction of these pollutants into ecosystems and bodies of water poses a threat to aquatic biota, as well as to plants and animals. Wastewater is the most common place for the detection of HMIs containing Sn^2+^, Cu^2+^, Fe^2+^, Zn^2+^, Na^1+^, and Co^2+^ [1]. Considering the distinctive interference or binding nature of other heavy metal ions, it is fascinating to observe their presence in a biological solution. The current hot topic investigates the synthesis of fluorescent sensors due to their widespread usage in biological and environmental applications [2]. These sensors can detect various metal ions. Consequently, there is an immediate need for advanced research that employs very sensitive analytical techniques to identify even trace amounts of heavy metals [3]. Because of its high resolution, improved sensitivity, and superior selectivity, spectrofluorometric analysis has been regarded as a dependable approach for the detection of heavy metal ions [4]. Although metal ions can be identified using ion exchange chromatography, electrochemistry, and colorimetric techniques but these analytical procedures are expensive, time-consuming, and challenging to use in real-time [5]. In comparison to more traditional methods, fluorescence analysis has several benefits, including great performance, low cost, rapid response, and extraordinary sensitivity [6]. Because of their usefulness, enhancement and quenching which allows them to be quantified based on the quantity of analytes that induce a change in fluorescence, making them extremely important. Furthermore, because a strong and pointed emission spectrum is chosen, metal ion responses produce unique fluorescence [7]. Extensive stud on the detection of various analytes has focused on ultraviolet–visible (UV–Visible) spectrophotometry. The UV–Visible technique is suitable for sensor binding because of these compounds' absorption band [8]. Due to its great efficiency and low cost, ultraviolet–visible spectroscopy is an excellent choice for detecting heavy metal ions, even if it is not as selective as the fluorescence approach. Examining metal ions with inexpensive organic molecules is possible using UV–Visible spectroscopy techniques. Using sunblock and other aesthetic filters can reduce health issues caused by exposure to solar ultraviolet (UV) radiation [9]. Due to its significant UV absorption, DSD has been utilized as a substance to block sunlight. However, because the stratum corneum can absorb some chemical substances that absorb UV light, using them in high concentrations may be dangerous. Because DSD has the ability to convert a portion of solar energy into fluorescent blue light, which gives the appearance of stunning whiteness, it is also frequently employed as a fluorescent whitening agent in paper, textile, plastic, and household detergents [10,11].

**Fig. 1:**
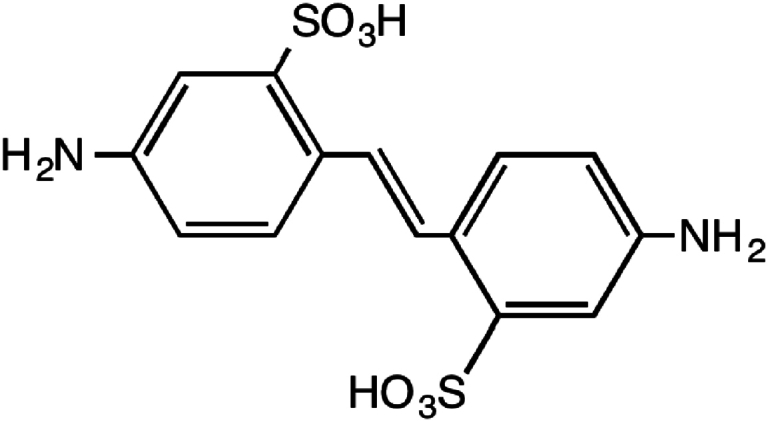
Chemical structure of DSD.

Optical brighteners, fluorescent whiteners, and dyes are all made from diamino-2,2′-stilbenedisulfonic acid (DSD) Fig. 1. Because of its estrogenic properties, DSD acid (also called Amsonic acid) may have caused impotence in industrial workers who were exposed to it. F344/N rats fed either 12,500 or 25,000 ppm amsonic acid for two years did not acquire cancer, according to the results of the feed tests. Both male and female B6C3F1 mice 11 showed no signs of carcinogenic activity when exposed to concentrations of 6250 or 12,500 ppm of this chemical [12]. DSD is an essential intermediary in both dyes and fluorescent brighteners. Fluorescent brighteners require higher quantities of this acid. One kind of azo dye is produced when 4-nitrotoluene-2-sulfonic acid or 4,4′-dinitro-2,2′-stilbenedisulfonic acid is combined with an alkaline environment. 4,4′-diamino-2,2′-stilbenedisulfonic acid tetrazo is used to create the colors, which are typical azo dyes [13].

Cancer is the top cause of mortality for individuals under 85 years old and is also the most common cause overall. It is predicted that there would be 611,720 cancer-related fatalities and 2,001,140 new cancer cases in the US in 2024 [14,15]. According to the global cancer incidence and death report, 14.1 million people will get a cancer diagnosis in the future years. An estimated 8.2 million people died from cancer, whereas 32.6 million People will have a chronic cancer illness [16]. By 2030, it's expected that cancer will kill 17 million people and cause 26 million new cases to be diagnosed [17]. According to Mortezaee et al. (2019) [18], even though a lot of people are working to find treatments, cancer is still the leading cause of death worldwide. In clinical trials, new synthetic chemotherapeutic drugs have fallen short of expectations despite their actual development costs [19]. There is an ongoing demand for practical and cost-effective anti-cancer medications due to the rising number of cancer patients. Fluorescence imaging is very important in many areas of modern research. The use of 4,4′-diamino-2,2′-stilbenedisulfonic acid (DSD), a well-known fluorophores for fluorescent materials, effectively inhibit the growth of various cancer cells, including those from the liver, ovary, breast, lungs and stomach [20,21]. Several mechanisms are attributed to DSD's anti-cancer actions. These include cell killing, regulating signal pathways, reducing oncogene expression, inhibiting P-gp channels, and limiting angiogenesis [22]. Since it generates inflammatory mediators, inflammation exacerbates a variety of disorders, including rheumatoid arthritis and ulcers [23]. After doing some investigation, DSD reduces inflammation by blocking the binding sites of nuclear factor kappa B (IkB), proinflammatory cytokines, and ATP [24,25]. To find multifunctional medications, DSD molecule was evaluated for antioxidant, anticancer, antibacterial, and enzyme inhibition (anti-Alzheimer and anti-inflammatory) properties. his was carried out since multiple investigations have demonstrated a close relationship between Alzheimer's disease, oxidative stress, inflammation, and cancer [26].

Antioxidants are a form of defensive mechanism that shields a body's internal organs from the oxidative processes that might be harmful due to reactive oxygen species (ROS). Along with being produced naturally in cells during stress and respiration, reactive oxygen species (ROS) have also been connected to radiation, bacterial and viral toxins, alcoholism, smoking, and psychological and emotional stress [21].

## 2 Material and methods

### 2.1 Experimental details

All the chemicals used in the current research were purchased from Sigma–Aldrich.

### 2.2 DSD solution preparation

In order to conduct the experiment, an aqueous solution of 5 × 10^−5^ M concentration of DSD was prepared. A concentration of 5 × 10^−5^ M of metal ions solutions was also prepared in double distilled water. In the cuvette, 2 mL of DSD solution and 10–100 μL of metal ion solution were measured. The solutions were examined using UV–Visible and fluorescence spectroscopic analysis [27].

### 2.3 Detection of metal ions by sensors

The fluorescence emission spectra of the sensors were recorded upon exposure to various metallic ions, including Sn^2+^, Cu^2+^, Fe^2+^, Zn^2+^, Na^1+^, Co^2+^, Bi^2+^, K^1+^, and Pb^2+^. The wavelength at which the DSD was excited was 450 nm. Using a UV-1800 spectrophotometer, absorption spectra of the sensor was recorded with competing metallic ions [28].

### 2.4 Spectroscopic studies

For the sensors, a 5 × 10^−5^ M solution was made with distilled water. The metal ion solutions of 1.5 × 10^−5^ M concentration were also prepared using double distilled water. 2 mL of a sensors solution were placed in experimental cuvettes at concentrations ranging from 10 to 100 μL, while solutions of metals with the same concentrations (10–100 μL) were examined in turn. The solutions were subjected to UV–Visible analysis. The sensors' and metal ions' fluorescence emission spectra were measured using a 1200 nm/min scanning rate on a Shimadzu spectrofluorometer. The spectrofluorometer was used to establish that the sensors' excitation wavelength was 450 nm. A Shimadzu UV-VIS-1800 spectrophotometer with a quartz path length of 1 cm was used for the UV–Visible investigation of the sensors containing metallic ions [10,29].

### 2.5 Fluorescence analysis

It is crucial to find and use a fluorescence probe that can sense and identify various metal ions. Finding metal ions that are compatible with the ions that are already in the premises is significant. The emission spectra of the sensors fluorescence altered noticeably as the concentration of Sn^2+^ ions increases. The response of DSD towards Sn^2+^ ions in the presence of different metal ions was investigated, including Cu^2+^, Fe^2+^, Zn^2+^, Na^1+^, Co^2+^, Bi^2+^, and K^1+^ by using UV–Visible, fluorescence spectrophotometer and pH meter analysis [30].

### *2.6* *In-vitro* assays

#### 2.6.1 Anti-cancer assays

##### 2.6.1.1 The MTT cell viability test for anticancer efficacy

At 37 °C in a humidified environment with 5 % CO_2_, the mouse embryonic fibroblast NIH/3T3 cell line was grown in DMEM media that was supplemented with 10 % FBS and antibiotics (50 units/ml penicillin and 50 units/ml streptomycin). The MTT test was used to assess the cytotoxicity of the samples against cultivated NIH/3T3 cells. Initial seeding density of 8.0 × 10^1^ cells/well in 200 μl media was used to seed NIH/3T3 cells into 96-well plates, which were then incubated for 24 h [31]. Afterwards, 200 μl of media containing samples at successive dilutions ranging from 0.0625 to 1 mg/mL was added to the removed culture medium. The cells were cultured for an additional day with cells that were just exposed to medium as a control group. Each well was then supplemented with 20 μl of MTT solution (5 mg/mL) in PBS. Carefully removing the medium containing the unreacted dye was done after incubation the cells for 4 h. Dimethyl sulfoxide (DMSO) was used to dissolve the resulting purple formazan crystals in 200 μl per well, and the absorbance was measured at 570 nm using a microplate spectrophotometer reader. As positive controls, the identical concentrations of etoposide and doxirubicin were used [32,33]. To determine the level of cell growth inhibition, the following formula was utilized;

$$\text{Cell}\;\text{viability}\;\%=\frac{\text{Mean}\;\text{of}\;\text{absorbance}\;\text{value}\;\text{of}\;\text{treatment}\;\text{group}}{\text{Mean}\;\text{of}\;\text{absorbance}\;\text{value}\;\text{of}\;\text{control}}\times 100$$

##### 2.6.1.2 Cytotoxicity study on HeLa cell line

The MTT (3-[4, 5-dimethylthiazole-2-yl]-2, 5-diphenyl-tetrazolium bromide) colorimetric test was used to study the cytotoxic potentials of pure substances on 96-well flat-bottomed micro plates [34]. In a nutshell, cervical cancer HeLa cells were grown in 75 cm^2^ flasks in a 5 % CO_2_ incubator at 37 °C in Minimum Essential Medium Eagle (MEME) supplemented with 5 % fetal bovine serum (FBS), 100 IU/mL of penicillin, and 100 μg/mL of streptomycin. A specific medium was used to dilute the exponentially developing cells after they were extracted and counted using a hemocytometer. A 96-well plate was used to inject 100 μL of cell culture at a concentration of 6x104 cells/ml. Following the incubation period of one night, the medium was drained and 200 μL of new media containing compounds ranging from 1 to 30 μM was introduced. Each well was supplemented with 200 μL of MTT (0.5 mg/mL) and left to incubate for an additional 4 h after 48 h. Later on, 100 μL of DMSO was injected to every well simultaneously. Using a micro plate reader (Spectra Max plus, Molecular Devices, CA, USA), the absorbance at 570 nm was measured to determine the degree of MTT reduction to formazan inside cells. The IC_50_, or concentration at which 50 % growth inhibition occurred in HeLa cells, was used to quantify the cytotoxicity [35]. We used the following formula to get the inhibition percentage:

Inhibition percentage = 100 minus the product of the following: (mean of optical density of test compound - mean of optical density of negative control) divided by (mean of optical density of positive control - mean of optical density of negative control)*100. Soft-Max Pro software (Molecular Device, USA) was used to process the findings (% inhibition).

##### 2.6.1.3 Cytotoxicity study on MCF-7 cell line

Overnight, MCF-7 cells from Manassas, VA, USA were placed in 96-well plates and kept in a CO2 incubator at 37 °C in DMEM with 0.5 μg/mL of hydrocortisone added. The insulin concentration was set at 10 μg/mL and the human epidermal growth factor concentration at 0.8 × 105 cells/mL. The cells were then exposed to varying concentrations of tested sample for 24, 48, and 72 h, after which a 5 mg/mL solution of MTT was added [36]. A wavelength of 570 nm was used to measure absorbance after incubating the cells (BioTek Instruments, Winooski, VT, USA).

#### 2.6.2 Anti-cholinesterase assays

##### 2.6.2.1 Acetyl and butyrylcholinesterase assays

DSD was compared to the galantamine reference in terms of its ability to inhibit acetyl and butyrylcholinesterase. The tested compound was prepared in DMSO at varying doses (500, 250, 125, 62.25, 31.25, 15.625, 7.81, 3.91 and 1.95 μg/mL). Iodide solutions of Acetylthiocholine iodide (AChEI) and butyrylthiocholine iodide (BChEI) were also prepared at a concentration of 0.05 mM. A phosphate buffer (pH 8) was used to dilute the enzymes. A solution of 5,5′-dithiobis-(2-nitrobenzoic acid) (DTNB) and distilled water containing 0.05 mM of acetyl and butyrylthiocholine iodide each was prepared and maintained at 8 °C for 15 min. After mixing the enzyme solution (5 μL), tested compound (205 μL), and DNTB (5 μL) solution, they were incubated at 30 °C for 15 min. A wavelength of 412 nm was used to test the mixture's absorption. The negative control consisted of all solutions devoid of test chemicals, whereas the positive control consisted of galantamine. The experiments were repeated in triplicate [37,38].

#### 2.6.3 Anti-inflammatory assays

##### 2.6.3.1 Anticyclooxygenase assay (COX-2)

The DSD was evaluated in relation to the COX-2 enzyme. The enzyme was placed on ice for 5 min for activation (300 units/mL). In a Tris-HCl buffer (pH 8), a 50 μL co-factor was added to the active solution. This co-factor included glutathione, hematin, 1.0 mM, and *N,N,N′,N′*-tetramethyl-*p*-phenylenediamine (TMPD). The blended solutions, which included 20 μL of tested compound and 60 μL of activated enzyme solution, were kept at room temperature for 5 min. The enzyme inhibition reaction was initiated by adding 20 μL of arachidonic acid [39]. The absorbance was measured at 570 nm after the reaction had been incubated for 5 min. The experiments were repeated in triplicate. Celecoxib, was used as standard drug in this assay [40].

##### 2.6.3.2 Anti 5-lipoxygenase assay (5-LOX)

The described protocol [41] was slightly modified to conduct this assay using linoleic acid as the substrate and montelukast as the standard drug. After dissolving the tested sample in phosphate buffer (pH 6.3), the enzyme solution (10,000 unit/μL) was added. After that, mixture was allowed to sit at room temperature for 5 min. After waiting 5 min, the absorbance at 234 nm was recorded. Triplicate runs of each experiment allowed for the calculation of inhibition percentages and IC_50_ values [42].

#### 2.6.4 Antioxidant assay

Using DPPH and ABTS free radical, we tested the DSD for their antioxidant properties *in-vitro*. Based on a previously published protocol [43]. After dissolving 20 mg of DPPH in 100 mL of methanol, a DPPH solution was formed. Using a UV–visible spectrophotometer, the absorbance was measured at 517 nm. Then, different quantities of the samples, ranging from 500 to 1.95 μg/mL, were mixed with 2 mL of DPPH solution. We incubated the sample/DPPH combination for 15 min at room temperature in the incubator [44]. The tests were carried out in triplicate. Following formula was used to find the percentages of DPPH free radicals inhibiton;

Percent Inhibition = Absorbance of control - Abs of sample/absorbance of control x 100

Similarly for the ABTS free radical scavenging assay, a mixture of 2.45 mM K_2_SO_4_ and 7 mM ABTS was prepared and mixed for the *in-vitro* ABTS test. The mixture was left to sit in darkness for 2 h. The ABTS cation solution was diluted with phosphate buffer (0.01 M, pH 7.4), and the absorbance value (0.70) was set at 734 nm. The test compound was mixed with 3.0 mL of ABTS solution at various doses (500, 250, 125, 62.25, 31.25, 15.625, 7.81, 3.91 and 1.95 μg/mL). Six minutes after the first minute, there was a noticeable drop in absorption at 734 nm. Ascorbic acid was used as positive control in this assay. The scavenging activities (%) and IC_50_ values were determined after three repetitions of the tests [45,46].

#### 2.6.5 Anti bacterial activity of DSD

##### 2.6.5.1 Media preparation

For the medium, we followed the directions on the label for Mueller-Hinton broth (MHB; BBLTM, France) and Mueller Hinton agar (MHA; DifcoTM, France). After being sterilized at 121 °C for 15 min, the media were placed in a sterile environment and kept at room temperature until they were needed again [47].

##### 2.6.5.2 Process for bacteria preparation

*Bacillus subtilis* FNCC 0059 and *Staphylococcus aureus* FNCC 0047 were among the Gram-positive bacteria used, while *Salmonella typhimurium* and *Escherichia coli* were among the Gram-negative bacteria sourced from the Food and Nutrition Culture Collection (FNCC) of Bacha Khan University Charsadda microbiology department. The bacterial stock was used to extract up to 20 mL of bacteria, which were then grown in NA medium. For many hours, the culture was kept at a temperature of 37 °C. After being revived, the bacterial colonies were tested for their antimicrobial properties [48].

##### 2.6.5.3 Making the McFarland 0.5 profile

From pure bacterial isolates, three or five colonies were chosen using a sterile inoculation loop. These were then injected into 3 mL of Mueller-Hinton broth (MHB) in a sterile tube and well mixed. Commercially produced 0.5 McFarland turbidity standards (bioMerieux® SA, France) were used to equal bacterial suspension turbidity levels. Using a spectrophotometer, we verified that the bacterial solution was accurate. For the purpose of comparison, sterile MHB was used and its absorbance was measured at a wavelength of 625 nm. Bacteria concentrations in MHB were measured using absorbance values between 0.08 and 0.10, corresponding to 107 to 108 per milliliter.

##### 2.6.5.4 Test for the susceptibility of disc diffusion

To make a solution with a concentration of 10 mg/mL, the samples were dissolved in 60 % methanol and then mixed vigorously using a vortex. Before layering the bacterial inoculum on top of the MHA plate, the inoculum was adjusted according to the McFarland 0.5 standard. A micropipette was used to extract 0.1 mL of solution from each inoculum. After dissolving the sample (20 mL) into a commercial blank disc (Oxoid, UK) at a concentration of 10 mg/mL, the disc's diameter was measured at 6 mm. Using sterile forceps, the disc was then put in the bacterial region for measurement. As a further step, 20 mL of 60 % methanol was impregnated into blank discs to serve as a negative control, and 10 mg of Amoxicillin was impregnated into the commercial antibiotic disc to serve as a positive control. The plates were kept at 37 °C for 24 h, and then the inhibition zones were measured. There was a duplicate run of each test set.

##### 2.6.5.5 Testing for minimum inhibitory concentration (MIC)

MIC The dilution method was also used to tested sample. Various concentrations of the examined substances were added to MHB medium with 0.5 MacFarlands Standard, and the bacterial cultures were allowed to complete one full loop of the exponential phase. The last step was a 24-h incubation period at 37 °C. Following the incubation time, the turbidity was assessed. A minimum inhibitory concentration (MIC) is the concentration of an extract at which all tested microorganisms are killed [49].

**Fig. 2:**
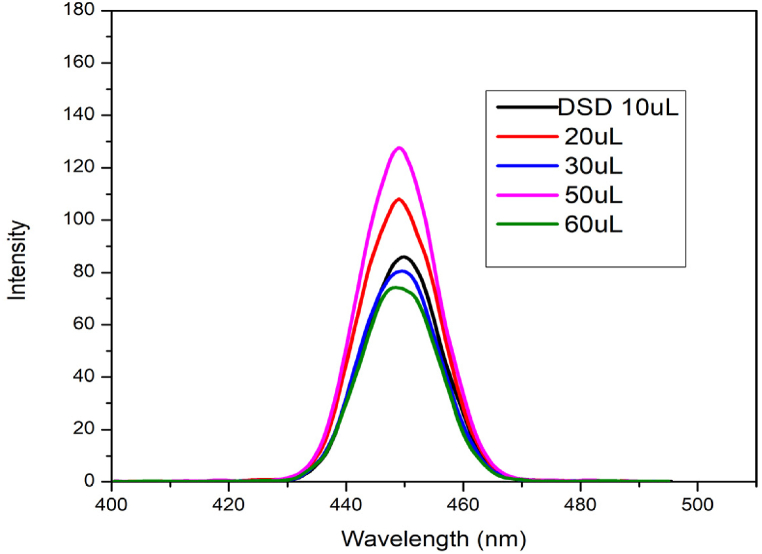
Fluorescence emission spectrum of sensor-DSD.

## 3 Results and discussion

### 3.1 Fluorescence spectra of 4,4′-diamino-2,2′-stilbene disulfonic acid (DSD)

The excitation wavelength of DSD fluorescent probe was chosen to be 450 nm. In order to determine their binding capabilities, the fluorescence spectrometer was used to tune the concentration and highly improved spectrum. This was accomplished by adding different concentrations of probe to 2 mL of solvent in a cuvette. when seen in Fig. -2, the fluorescence emission spectra progressively improved when more probe was added. According to Fig. -2, the intensity of the fluorescence emission keeps rising when the concentration of the sensors is raised little by little. The optimization was achieved by adding 50–60 μL of sensors, after which the improvement ceased. The Chelation Enhanced Fluorescence Effect (CEFE) is responsible for the improvement of fluorescence emission spectra Fig. 2.

### 3.2 Binding behavior of stannous chloride

**Fig. 3:**
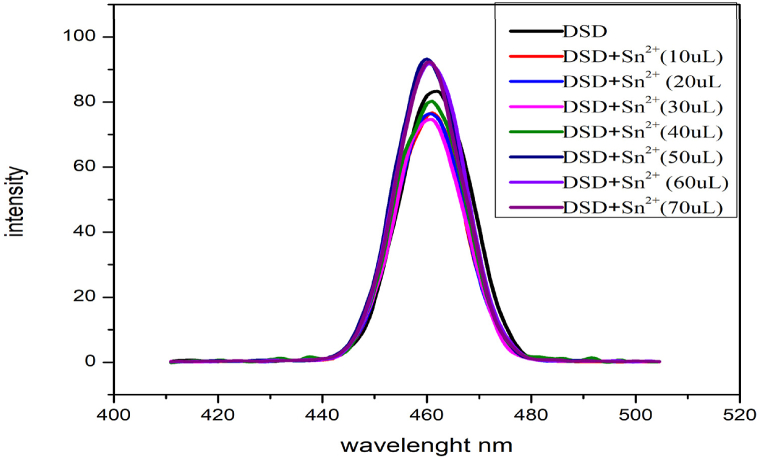
Fluorescence emission spectrum of Sn^2+^.

Detecting Sn^2+^ ions in water at nanomolar levels is possible using the fluorescent probe DSD. Initial observations were made of the compound's emission spectra. Adding 10 μL of Sn^2+^ ion increased the peak and changed the fluorescence spectra. The peak was further enhanced when 20uL of Sn^2+^ was added further, causing a little shift. As additional metal ion solution was added, the peak progressively diminished due to quenching. The increase happened as a result of the chelation improved fluorescence effect and the quenching effect was noticed since the compound is a powerful reducing agent Fig. 3.

### 3.3 Mechanism of fluorescence sensing

**Fig. 4:**
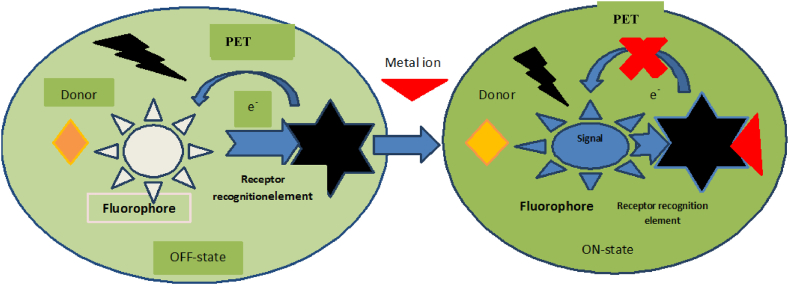
Schematic depiction of a photo induced electron transfer (PET) mechanism-based fluorescence sensor and its potential operational mode.

When metal ions bind to sensors, they may dampen or amp up the intensity of fluorescence emission. The chelation effect has the potential to either increase the emission spectrum of fluorescence (the Chelation Enhanced Fluorescence Effect) or quench it (the Chelation Enhancement Quenching Effect). The connection of the bathochromic and hypsochromic emission spectra is possible as a consequence of both processes. The intensity of fluorescence emission is enhanced when Sn^2+^ ions undergo a photo-induced electron transfer pathway. This happens because the coordinating chemical donates an electron pair to the excited orbital of the fluorophores, which causes the fluorophores to undergo this process. In order to turn on the fluorescence spectra, metal ions reduce the lone pair energy of the sensors during coordination, blocking the Photo-Induced Electron transfer process Fig. 4.

### 3.4 Comparison of various metallic ions fluorescence emission spectra

**Fig. 5:**
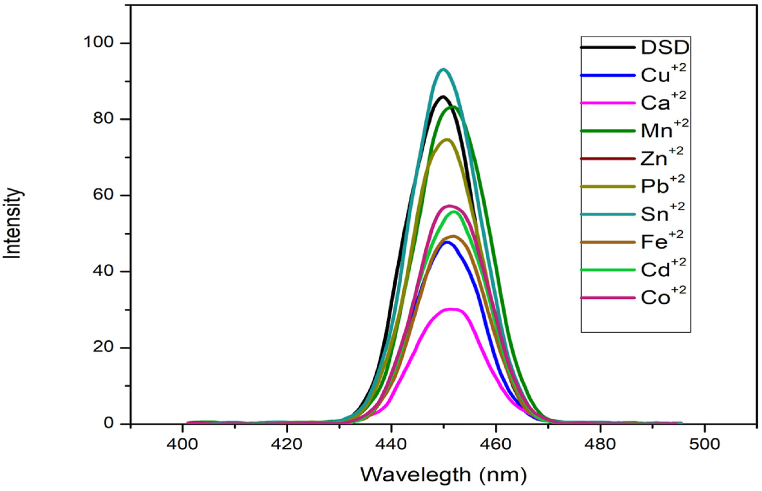
Fluorescence emission spectra of DSD with various metal ions.

To evaluate the sensing capabilities of fluorescent probes based on organic molecules, selectivity is of the highest importance. In the presence of suitable metallic cations, these probes have been able to detect Sn^2+^ ions. As the concentration of Sn^2+^ ions was gradually increased or decreased, the fluorescence emission spectra (FES) of the ions varied dramatically in tandem with sensor-DSD. Fluorescent probes with specificity for Sn^2+^ have been investigated in this work. Despite the presence of Sn^2+^, Cu^2+^, Fe^2+^, Na^1+^, Co^2+^, Bi^2+^, and K^1+^ ions, sensor-DSD is able to detect only Sn^2+^ in the presence of Pb^2+^, Cu^2+^, Fe^2+^, Zn^2+^, Na^1+^, Co^2+^, Bi^2+^, and K^1+^ ions without inhibition. Sn^2+^ spectra were compared to those of the compatible metallic cations as shown in Fig. 5.

### 3.5 UV–visible spectrophotometric studies

‘ Optical spectrophotometry was used to further develop the sensing behavior of the organic fluorescence sensor DSD towards metallic ions. The UV spectra of DSD showed prominent absorption bands at 350 nm. The inclusion of Sn^2+^ ions into the sensor has reduced the intensity of the DSD band. Under the optical spectrophotometer, the DSD sensor's spectra remained unchanged when exposed to ions of Pb^2+^, Cu^2+^, Fe^2+^, Na^1+^, Co^2+^, Bi^2+^, and K^1+^, among others. This research shows that, even when other, perhaps competing metal ions are present, the DSD sensor only picks up on Sn^2+^ ions. As the quantity of Sn^2+^ ions increased, the isosbestic point at 350 nm of the sensor –DSD decreased. Interpretive behavior, rather than the participation of other metal ions in the premises, was shown by the relative absorbance of the sensor DSD for Sn^2+^ ions.

### 3.6 UV–visible spectra of DSD

The binding ability of the sensor-DSD was studied using a UV–Visible spectrophotometer. The UV–Visible spectra of DSD exhibiting a significant absorption band at 350 nm. Three milliliters (3 mL) was the optimum and required concentration. When metal ions were treated with the optimal concentration of DSD, the required absorption band was obtained and the sensor's concentration was noted. The absorption of the band was progressively raised with the incremental addition of sensor DSD.

### 3.7 Binding behavior of stannous chloride

**Fig. 6:**
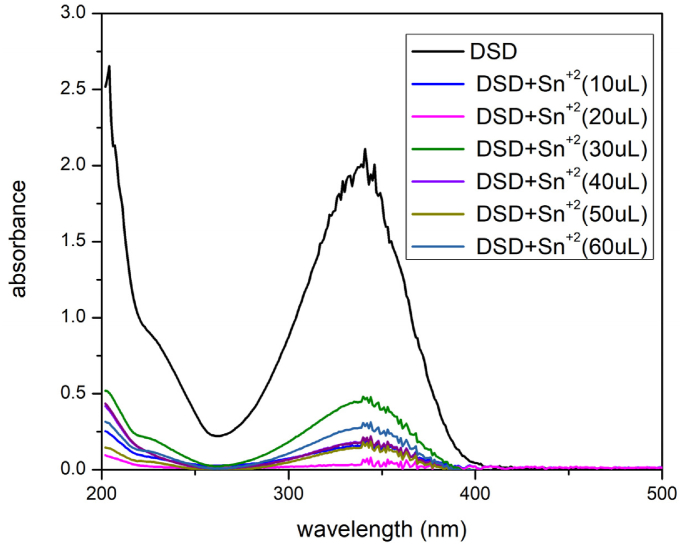
DSD with tin chloride UV–Visible spectra.

UV–Visible spectroscopy revealed significant absorption bands, which indicated the sensor's ability to bind Sn^2+^. The band at 350 nm initially showed substantial absorption when 3 mL of the stock solution was added. Initially, in order to enhance the intensity of the band, 10uL of Sn^2+^ ion solution was added. The bands' intensity decreased with the addition of 20uL. The intensities were further reduced with the addition of 30, 40, and 50 μL Fig. 6.

### 3.8 Comparison of various metallic ions UV–visible spectra

**Fig. 7:**
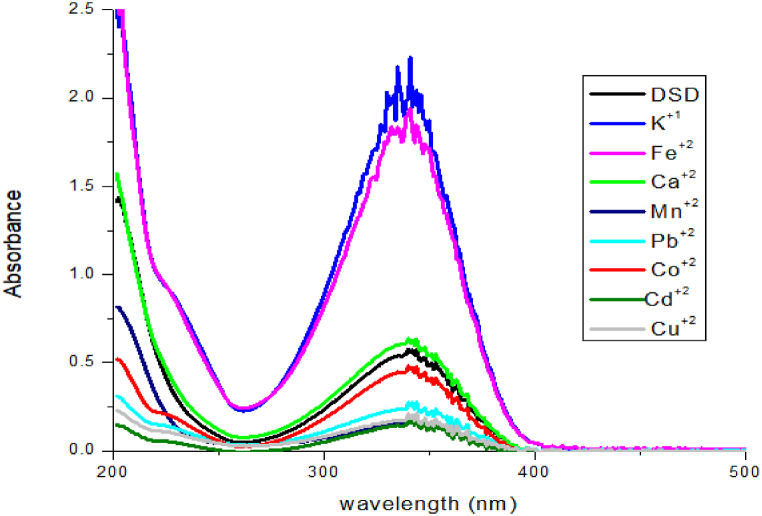
UV–Visible absorption spectrum of sensor DSD along with metal ions.

Utilizing UV–Visible spectrophotometry, the binding capabilities and comparative studies of DSD with all metals (Sn^2+^, Cu^2+^, Fe^2+^, Zn^2+^, Na^+1^, Co^2+^, Mn^2+^, K^+1^) was further investigated. The absorption spectra of DSD in aqueous environments containing metal cations are shown in Fig. 5. The intensity of the band 350 nm has been diminished due to the incorporation of Sn^2+^ into the sensor (DSD). Using sensor DSD, no optical changes were seen for other metal ions such as Pb^2+^, Cu^2+^, Fe^2+^, Zn^2+^, Na^+1^, Co^2+^, Mn^2+^, K^+1^ ions. It is evident that DSD is capable of selectively detecting Sn^2+^ even when other metal cations are present. The corresponding absorption bands for DSD diminish with the increasing addition of Sn^2+^ ions, reaching an isosbestic point at 350 nm. Sn^2+^ ions were shown to be selectively absorbed by a DSD that was loaded with all metal cations, as opposed to other metal ions Fig. 7.

### 3.9 Effect of pH

To study the effect of pH on DSD acid's binding capacity with metal ions, fluorescence spectroscopy was used. The fluorescence at 350 nm for the DSD-Sn^2+^ complexes spiked at pH 5.0 and remained constant up to pH 9.5. This suggests that deprotonation is a mechanism involved in the creation of DSD-Sn^2+^ complexes. Additionally, we investigated how pH impacted the production of DSD with every other metal. The fluorescence quenching band at 350 nm in DSD acid remained constant over a pH range of 4–8. The intensity at 350 nm increased when pH level increased over 8. This results from the dissociation of DSD treating with other metal ions. Indicating that behavior of DSD with other metal ions complexes do not form in this pH range, absorbance was essentially nonexistent at pH values lower than 4.

### 3.10 Anticholinesterase assay results

As can be seen in Table 1 for AChE and BChE, respectively, the isolated molecule was tested for anticholinesterase potentials using Ellman's assay. The AChEI potentials of compound (1) were exceptional at the 500, 250, 125, 62.5, 31.25, 15.625, 7.81, 3.91 and 1.95 μg/mL concentrations that were examined. At a concentration of 1000 μg/mL, DSD inhibited acetylcholinesterase the most effectively, with an activity of 86.04 ± 1.52 % and an IC_50_ of 12.18 μM. As the concentration was reduced from 500 μg/mL to 31.25 μg/mL, the percentage of inhibitions decreased to 82.56 ± 1.80, 78.64 ± 1.50, 72.74 ± 1.02, and 67.23 ± 0.84 %, respectively. The standard medication galanthamine inhibits AChE at doses of 1000, 500, and 250 μg/mL, respectively, with IC_50_ values of 8.60 μM with percent inhibition of 94.22 ± 0.01, 92.28 ± 0.43, and 85.35 ± 0.83 %. DSD's butyrylcholinesterase inhibitory effects (BChEI) are listed in Table 1. DSD inhibited 82.12 ± 0.34, 77.45 ± 1.67, 73.56 ± 0.52, 68.76 ± 0.70, and 63.33 ± 1.31, 56.45 ± 0.41, 49.87 ± 0.89, 43.63 ± 0.11 and 37.45 ± 1.07 % BChE at doses of 1000 to 1.95 μg/mL, respectively. Its IC_50_ value was 20.87 μM. The standard drug galantamine exhibited 91.76 % inhibition at highest concentration causing IC_50_ 9.78 μM.

**Table 1:** Anti-cholinesterase enzyme inhibitions of the compound DSD.

Comp/Standard	Conc (μg/mL)	AChE Percent Inhibition (mean ± SEM)	IC_50_ (μM)	BChE Percent Inhibition (mean ± SEM)	IC_50_ (μM)
**DSD**	500	86.04 ± 1.52***	12.18	82.12 ± 0.34***	20.87
	250	82.56 ± 1.80***		77.45 ± 1.67***	
	125	78.64 ± 1.50***		73.56 ± 0.52***	
	62.50	72.74 ± 1.02***		68.76 ± 0.70***	
	31.25	67.23 ± 0.84***		63.33 ± 1.31***	
	15.625	62.34 ± 0.30**		56.45 ± 0.41***	
	7.81	55.12 ± 0.80**		49.87 ± 0.89***	
	3.91	48.46 ± 1.10*		43.63 ± 0.11***	
	1.95	41.89 ± 0.77**		37.45 ± 1.07***	
**Galantamine**	500	94.67 ± 0.55	8.70	91.76 ± 0.56	9.78
	250	89.55 ± 1.49		86.87 ± 0.99	
	125	85.88 ± 2.65		83.89 ± 1.75	
	62.50	78.68 ± 1.94		79.54 ± 0.52	
	31.25	75.54 ± 2.60		75.34 ± 0.22	
	15.625	68.66 ± 0.46		67.67 ± 0.11	
	7.81	63.34 ± 0.12		62.56 ± 0.50	
	3.91	54.82 ± 0.80		51.23 ± 0.21	
	1.95	48.37 ± 0.31		45.90 ± 0.18	

Data is represented as mean ± SEM, n = 3; Two-way ANOVA followed by Bonferroni test was applied for significant difference between standard drugs and test samples at 95 % confidence interval. ***P < 0.001 as comparison to the standard drug.

### 3.11 Anti-inflammatory assay results

To determine if the substances under investigation have anti-inflammatory properties, the *in-vitro* cyclooxygenase assay is often used. Researchers have proposed employing *in-vitro* assays to determine the anticipated levels of COX inhibition of the test chemicals since they are rapid and easy compared to clinical research [50]. Inflammation is mostly caused by the COX-2 enzyme. This inducible enzyme produces mediators that cause inflammation. While COX-2 is almost nonexistent in most tissues under normal conditions, its expression may be ten to eighty times higher in inflammatory conditions or after exposure to mutagenic stimuli. Most pharmaceutically accessible NSAIDs work by blocking both constitutive and inducible COX-2, which in turn inhibits the formation of prostaglandin (PG) and thromboxane (TX). Inhibition of COX-2 may account for the anti-inflammatory, analgesic, and antipyretic therapeutic actions of nonsteroidal anti-inflammatory drugs (NSAIDs) [51]. An evaluation was conducted to determine the molecule's capacity to inhibit the COX-2 enzyme. The compound's potency was determined by calculating its IC_50_ value in μg/ml, which is the concentration of the drug needed to inhibit the enzyme 50 %. Hydroperoxy metabolites (HPETEs) are produced by lipoxygenases (LOXs), which are dioxygenases that do not include heme iron. Molecular oxygen is incorporated into the substrate by these enzymes. In humans, six different LOX isoforms are functional. The second critical metabolic step that creates eicosanoids is initiated by the 5-lipoxygenase (5-LOX), an isozyme of LOX. These are found often in plants, fungi, and animals. Polymorphonuclear leukocytes (eosinophils and neutrophils) and mono-nuclear cells (lymphocytes, monocytes, and macrophages) represent the majority of myeloid cells that express the 5-LOX protein. The last result of the 5-LOX pathway, leukotriene B4, mediates a variety of allergic and inflammatory diseases, such as atherosclerosis, cancer, and cardiovascular illnesses. In contrast, reducing leukotriene levels, which are caused by COX-1/2 inhibitors, may help reduce the risk of cardiovascular and gastrointestinal disorders, respectively, decreasing 5-LOX. One interesting finding is that COX-2 and 5-LOX co-inhibition has the potential to reduce side effects on the gastrointestinal and cardiovascular systems while keeping the primary impact of COX-1/2 inhibitors [52,53]. In this assay the DSD exhibited 76.02 ± 1.44, 72.52 ± 1.34, 68.68 ± 1.78, 62.72 ± 1.08, 57.21 ± 0.80, 52.34, 46.78, 41.90 and 35.40 percent inhibition of COX-2 casing IC_50_ value 32.15 μM. Similarly the tested sample inhibit the 5-LOX as 72.10 ± 0.30, 67.49 ± 1.61, 63.51 ± 0.53, 58.78 ± 0.76, 53.31 ± 1.33, 48.89, 43.44, 36.89 and 31.90 with IC_50_ value 56.02 μM respectively as shown in Table 2. The standard drug celecoxib and montelukast shown excellent potential with IC_50_ 5.72 and 4.35 μM respectively.

**Table 2:** COX-2 and 5-LOX enzyme inhibitions of the DSD.

Comp/Standard	Conc (μg/mL)	COX-2 Percent Inhibition (mean ± SEM)	IC_50_ (μM)	5-LOX Percent Inhibition (mean ± SEM)	IC_50_ (μM)
**DSD**	500	76.02 ± 1.44***	32.15	72.10 ± 0.30***	56.02
	250	72.52 ± 1.34***		67.49 ± 1.61***	
	125	68.68 ± 1.78***		63.51 ± 0.53***	
	62.50	62.72 ± 1.08***		58.78 ± 0.76***	
	31.25	57.21 ± 0.80***		53.31 ± 1.33***	
	15.625	52.34 ± 0.30***		48.89 ± 0.99***	
	7.81	46.78 ± 0.56***		43.44 ± 0.22***	
	3.91	41.90 ± 1.11***		36.89 ± 0.11***	
	1.95	35.40 ± 1.02***		31.90 ± 1.12***	
**Celecoxib**	500	90.61 ± 0.57	5.72	_________	
	250	85.59 ± 1.47			
	125	81.85 ± 2.63			
	62.50	76.62 ± 1.90			
	31.25	71.53 ± 2.63			
	15.625	66.45 ± 1.11			
	7.81	61.56 ± 0.52			
	3.91	55.89 ± 0.93			
	1.95	49.90 ± 0.80			
**Montelukast**	500	_________		85.73 ± 0.53	4.35
	250			81.85 ± 0.95	
	125			77.87 ± 1.73	
	62.50			72.56 ± 0.52	
	31.25			68.32 ± 0.20	
	15.625			64.34 ± 0.30	
	7.81			58.54 ± 0.52	
	3.91			53.65 ± 0.67	
	1.95			48.23 ± 0.11	

The data is shown as the average plus or minus the standard error of the mean (n = 3). To compare the standard medications and test samples, a two-way ANOVA was conducted, followed by a Bonferroni test, with a 95 % confidence interval. ***P < 0.001 as comparison to the standard drug.

### 3.12 Anti-oxidant assay results

Cancer, inflammation, Alzheimer's disease, and diabetes are just a few of the illnesses that oxidative stress may accelerate. We now know that oxidative stress, which develops in our bodies (including the brain) as a result of an increase in free radical concentration, is the etiological cause of most disorders [54]. Neuronal abnormalities that eventually manifest as Alzheimer's disease may have their origins in free radicals. It has been suggested that free radicals have a role in the development of diabetes mellitus. Accordingly, the results of the DPPH and ABTS free radical scavenging assays show that the DSD can efficiently remove free radicals. The tested DSD displayed 81.58 ± 1.12, 77.65 ± 1.34, 74.90 ± 0.96, 69.03 ± 0.48, 65.90 ± 0.48, 61.56 ± 0.52, 56.34 ± 0.68, 51.64 ± 0.60 and 46.23 ± 0.11 percent inhibition against ABTS with IC_50_ 8.59 μM at the concentration ranging from 500 to 1.95 μg/mL. Likewise, in the DPPH assay the DSD exhibited 83.33 ± 0.49, 77.03 ± 0.23, 73.00 ± 0.58, 68.67 ± 0.89, 62.00 ± 1.15, 56.34 ± 1.30, 51.87 ± 0.89, 44.22 ± 0.20 and 36.99 ± 0.45 % inhibition with IC_50_ 20.54 μM. The standard drug displayed 3.87 and 8.12 μM IC_50_ against both of the free radical like ABTS and DPPH respectively as shown in Table 3.

**Table 3:** ABTS and DPPH free radical scavenging values of the DSD.

Comp	Concentration (μg/mL)	ABTS % Inhibition	IC_50_ (μM)	DPPH % Inhibition	IC_50_ (μM)
**DSD**	500	81.58 ± 1.12***	8.59	83.33 ± 0.49***	20.54
	250	77.65 ± 1.34***		77.03 ± 0.23***	
	125	74.90 ± 0.96***		73.00 ± 0.58***	
	62.5	69.03 ± 0.48**		68.67 ± 0.89***	
	31.25	65.90 ± 0.48***		62.00 ± 1.15***	
	15.625	61.56 ± 0.52***		56.34 ± 1.30***	
	7.81	56.34 ± 0.68***		51.87 ± 0.89***	
	3.91	51.64 ± 0.60***		44.22 ± 0.20***	
	1.95	46.23 ± 0.11***		36.99 ± 0.45***	
**Ascorbic acid**	500	95.85 ± 0.18	3.87	97.53 ± 0.20	8.12
	250	91.59 ± 0.30		93.62 ± 0.17	
	125	87.75 ± 0.14		88.42 ± 0.11	
	62.5	84.47 ± 0.49		84.20 ± 0.15	
	31.25	81.12 ± 0.34		81.35 ± 0.18	
	15.625	75.56 ± 0.52		73.87 ± 1.11	
	7.81	71.87 ± 0.81		67.45 ± 0.42	
	3.91	66.22 ± 1.20		62.98 ± 1.02	
	1.95	61.16 ± 1.02		55.34 ± 1.20	

The data is shown as the mean ± SEM, with n = 3. To determine whether there was a significant difference between the standard medications and test samples, a two-way ANOVA followed by a Bonferroni test was used, with a 95 % confidence interval. ***P < 0.001, **P < 0.01, *P > 0.05 as comparison to the standard drug ascorbic acid (AA).

### 3.13 Anticancer activity

When it comes to female cancers, breast cancer is both the most common and the second most deadly [55]. In order to determine whether the tumor grows or shrinks in response to hormonal therapies, radiation, chemotherapy, or all, the balance between proliferation and apoptosis is critical [56]. The death receptor mediated route and the mitochondrial pathway are two ways in which cell death might take place. When it comes to breast cancer, nevertheless, the mitochondrial route seems to be the most important [57]. Many human illnesses, including cancer and neurological disorders, are now believed to have apoptotic pathway abnormalities as a contributing factor. To test for cytotoxic action, we used the methyl thiazol tetrazolium (MTT) assay, a straightforward and dependable method that assesses cell viability. Various doses incubated with cancer cells to determine their viabilities. You may find the results of the sample's cytotoxicity against NIH/3T3, HeLa, and MCF-7 cell lines in Table 4. At concentrations of 500, 250, 125, 62.50, 31.25, 15.625, 7.81, 3.91 and 1.95 μg/mL, the substance under test exhibited cytotoxicity against NIH/3T3 cell lines with a result of 78.05 ± 1.33, 72.53 ± 3.87, 67.30 ± 0.92, 62.43 ± 1.20, and 57.02 ± 1.04, 35.23 ± 0.21, 53.45 ± 0.41, 46.65 ± 0.67 and 41.33 ± 0.66 % respectively. The NIH/3T3 cell lines were inhibited by DSD, which exhibited an LD_50_ value of 32.59 μM. When tested against HeLa cell lines at doses of 500, 250, 125, 62.50, 31.25, 15.625, 7.81, 3.91 and 1.95 μg/mL, the tested sample showed cytotoxicity of 87.38 ± 0.67, 81.76 ± 1.22, 76.45 ± 1.17, 71.41 ± 1.00, and 65.23 ± 2.29, 39.34 ± 0.30, 61.65 ± 0.69, 54.34 ± 0.32 and 45.65 ± 0.11 % respectively. When tested against HeLa cell lines, the LD_50_ value was 15.31 μM. In addition, at concentrations of 500, 250, 125, 62.50, 31.25, 15.625, 7.81, 3.91 and 1.95 μg/mL, the DSD exhibited 81.98 ± 1.56, 73.03 ± 0.76, 57.73 ± 0.66, 51.07 ± 0.47, and 45.45 ± 1.05, 23.67 ± 0.77, 41.78 ± 0.70, 34.65 ± 0.11 and 28.23 ± 0.19 % cytotoxicity against MCF-7 cells, respectively. According to Table 4, the LD_50_ value against MCF-7 cells was 96.46 μM.

**Table 4:** Research on the anti-cancer effects of chemical 1 in NIH/3T3, HeLa, and MCF-7 cell lines.

Sample	Conc. (μg/mL)	NIH/3T3	LD_50_ (μM)	HeLa	LD_50_ μg/ml	MCF-7	LD_50_ (μM)
**DSD**	500	78.05 ± 1.33	32.59	87.38 ± 0.67	15.31	81.98 ± 1.56	96.46
	250	72.53 ± 3.87		81.76 ± 1.22		73.03 ± 0.76	
	125	67.30 ± 0.92		76.45 ± 1.17		57.73 ± 0.66	
	62.5	62.43 ± 1.20		71.41 ± 1.00		51.07 ± 0.47	
	31.25	57.02 ± 1.04		65.23 ± 2.29		45.45 ± 1.05	
	15.625	53.45 ± 0.41		61.65 ± 0.69		41.78 ± 0.70	
	7.81	46.65 ± 0.67		54.34 ± 0.32		34.65 ± 0.11	
	3.91	41.33 ± 0.66		45.65 ± 0.11		28.23 ± 0.19	
	1.95	35.23 ± 0.21		39.34 ± 0.30		23.67 ± 0.77	

The results show the average plus or minus the standard error of three separate measurements. Doxirubicin, a standard medication, exhibited cytotoxicity against NIH/3T3, HeLa, and MCF-7 cells at 89.40, 92.00, and 88.53 % of the cells, respectively. Individually, the LD50 values for these cells were 15, 7, and 11 μg/mL.

### 3.14 Antibacterial activity

Now a day's the antimicrobial medications aren't very good, a new trend is emerging: germs that are resistant to antibiotics. New compounds to combat germs that are resistant to many drugs are the focus of researchers' efforts to find a solution to this problem. Here, the focus of the present research was on fighting infections caused by bacteria that are resistant to more than one antibiotic. Table 5 shows that the negative control did not have any visible zones of bacterial activity. According to Monks et al. [58], there is no inhibitory effect when the range of the inhibition zone is less than 7 mm. The DSD displayed 16.40, 17.70, 18.00 and 18.80 mm zone of inhibition against *Salmonella Typi, Escherichia coli, Sreptococcus aureus and Bacillus substilis*. The positive control drug amoxicillin displayed excellent zone of inhibition against all bacteria. Furthermore, after evaluation of the anti-bacterial activity the minimum inhibitory concentrations (MIC) values were also explored for that of the standard and tested samples.

**Table 5:** Gram-negative and Gram-positive bacterial inhibition zone.

Samples	Zone of inhibition (mm)			
	*Salmonella Typi*	*Escherichia coli*	*Sreptococcus aureus*	*Bacillus substilis*
**Negative Control**	0.00 ± 0.00	0.00 ± 0.00	0.00 ± 0.00	0.00 ± 0.00
**Amoxicillin**	30.00 ± 0.01	29.00 ± 0.07	35.40 ± 0.02	38.50 ± 0.06
**DSD**	16.40 ± 0.08	17.70 ± 0.05	18.00 ± 0.04	18.80 ± 0.05

Note: NC (−): sterile distilled water.

In order to determine the minimum inhibitory concentration (MIC), researchers looked at the lowest concentration at which the test bacteria were no longer able to proliferate. The solution color in the test tube seemed to be clear during a 24-h incubation period at a concentration of 500–31.25 μg/mL. We picked a concentration of 31.25 μg/ml as the MIC value since it was the lowest concentration of the tested samples that could induce an inhibitory zone on all Gram-positive and Gram-negative bacteria, as shown in Table 6. Hence, for *Salmonella Typi, Sreptococcus aureus, and Bacillus substilis*, the minimum inhibitory concentration (MIC) against both Gram-positive and Gram-negative bacteria was 125 μg/mL.According to Table 6, the minimum inhibitory concentration (MIC) against *Escherichia coli* was 250 μg/mL.

**Table 6:** Bacterial MIC values (Gram negative and Gram positive).

Samples	Conc.	MIC values (μg/mL)			
		*Salmonella Typi*	*Excherichia coli*	*Sreptococcus aureus*	*Bacillus substilis*
**Amoxicillin**	500	–	–	–	–
	250	–	–	–	–
	125	–	–	–	–
	62.5		+	–	+
	31.25	+	+	+	+
**DSD**	500	–	–	–	–
	250	–	–	–	–
	125	–	+	–	–
	62.5	–	+	–	–
	31.25	+	+	+	+

## 4 Conclusion

This work demonstrates the ability to selectively detect Sn^2+^ ions over competing metal ions using a commercially available fluorescent probe called DSD. This work shown that the DSD probe had a high specificity for detecting Sn^2+^ ions in water. It was possible to test the sensor's fluorescence capabilities since Sn^2+^ ions were present. We may attribute the improved fluorescence to the combination of stannous ions (Sn^2+^) DSD. Potentially, blocking the photo-induced electron transfer pathway (PET) is responsible for the increased fluorescence. Selective detection of metal ions from water or any other matrix for industrial applications is one of the possible future uses of biosensors that might be improved with the use of the right organic material throughout their development. In addition, further screening and evaluations of DSD are warranted, but based on the available data and ongoing research, it appears that DSD has bioactive properties and might be a promising drug runner for the treatment of cancer, inflammation associated with diabetes mellitus, and Alzheimer's disease.

## Funding

This research was funded by 10.13039/501100006261Taif University, Saudi Arabia, Project number. (TU-DSPP-2024-148).

## Data availability statement

Data will be made available on request.

## CRediT authorship contribution statement

**Madeeha Shabnam:** Investigation, Formal analysis. **Eman A. Alabdullkarem:** Methodology, Conceptualization. **Muhammad Saeed Jan:** Data curation, Conceptualization. **Saad H. Alotaibi:** Software, Resources. **Khairia Mohammed Al-Ahmary:** Visualization, Software, Resources. **Muhammad Ibrar:** Project administration, Investigation, Conceptualization. **Mohamed Hussien:** Validation, Methodology, Conceptualization. **Asmaa E. Sherif:** Funding acquisition, Formal analysis, Conceptualization.

## Declaration of competing interest

The author declares no known competing financial interests or personal relationships that could have appeared to influence the work reported in this paper.
